# Prolonged pain-to-balloon time still impairs midterm left ventricular function following STEMI

**DOI:** 10.1186/s12872-025-04484-3

**Published:** 2025-01-23

**Authors:** Dominik Felbel, Sabrina Fackler, Rachel Michalke, Michael Paukovitsch, Matthias Gröger, Mirjam Keßler, Nicoleta Nita, Yannick Teumer, Leonhard Schneider, Armin Imhof, Dominik Buckert, Wolfgang Rottbauer, Sinisa Markovic

**Affiliations:** Department of Cardiology, Ulm University Heart Center, Albert-Einstein-Allee 23, 89081 Ulm, Germany

**Keywords:** STEMI, Pain-to-balloon time, Left ventricular dysfunction

## Abstract

**Background:**

ST-elevation myocardial infarction (STEMI) demands near-time reperfusion to reduce the risk of long-term heart failure. This study evaluates the proportion of impaired left ventricular ejection fraction (LVEF) following STEMI in the context of current healthcare settings at a tertiary care center equipped with the most advanced and up-to-date standards of care.

**Methods:**

Patients experiencing STEMI as their first manifestation of coronary artery disease were analyzed, as these individuals had no prior experience with heart-related chest pain. LVEF was assessed by levocardiography at admission and semiautomatically using TOMTEC in patients with eligible full-cycle echocardiography of 2- and 4-chamber view available at discharge and 1-year follow-up (FU). Pain-to-balloon time was divided into quartiles (Q) [0-111, 112–159, 160–246 and 247–784 min]. Multiple logistic regression analysis identified independent predictors of reduced LVEF < 50% at 1-year FU.

**Results:**

A total of 1,379 consecutive STEMI patients were reviewed from 2010 to 2017, with 130 meeting the inclusion criteria. Mean age was 63 ± 12 years, 75% were male, 14% had diabetes, 72% had arterial hypertension, and 56% had history of smoking. LVEF was reduced in 94% of patients at admission, 69% at discharge, and remained reduced in 45% at the 1-year follow-up. Anterior wall myocardial infarction (OR 3.2 [95%-CI 1.2–6.9], *p* = 0.018) and increasing pain-to-balloon time across quartiles (Q2: OR 15.7 [95%-CI 1.8–140.4], *p* = 0.014; Q4: OR 33.7 [3.4–278.7] *p* = 0.002) were independently associated with reduced LVEF at 1 year.

**Conclusion:**

Despite optimal medical management and advanced healthcare structures, nearly half of patients with STEMI as their first presentation of coronary artery disease continue to exhibit reduced LVEF at 12-months. Anterior wall myocardial infarction and pain-to-balloon time exceeding 2 h remain independent predictors of left ventricular dysfunction. Further improvements in healthcare systems and public education are essential to reduce treatment delays and improve long-term outcomes.

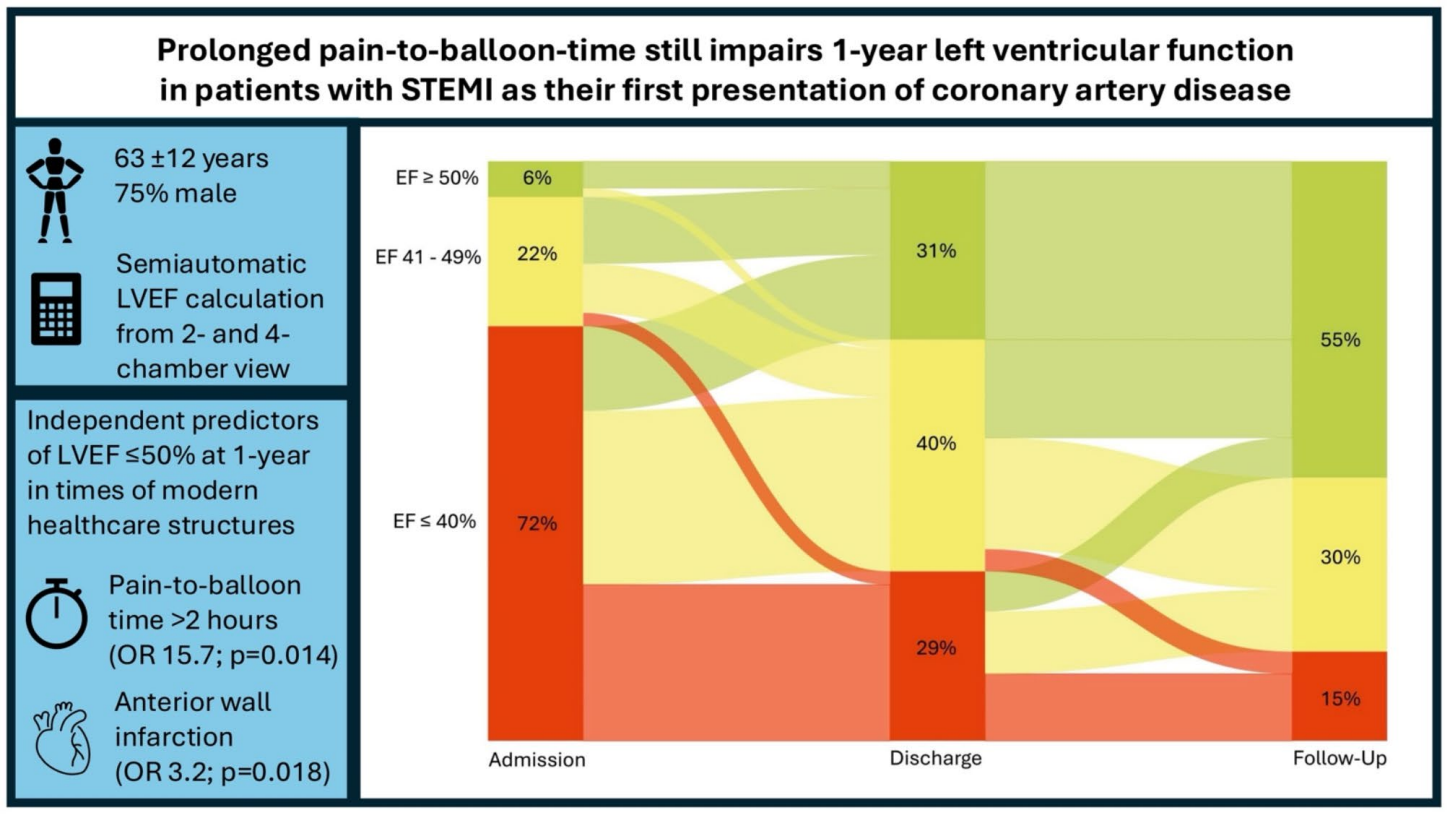

**Supplementary Information:**

The online version contains supplementary material available at 10.1186/s12872-025-04484-3.

## Introduction

Although left ventricular ejection fraction (LVEF) is known to recover in many patients following myocardial infarction, a significant proportion remains at risk of persistent left ventricular dysfunction and chronic heart failure [1, 2]. Besides myocardial infarction type and location, various patient and healthcare-structure related factors contribute to the risk of chronic heart failure and heart failure hospitalization [2, 3]. Comorbidities like arterial hypertension, diabetes, atrial fibrillation, chronic kidney disease and age were associated with an elevated risk of developing chronic heart failure following myocardial infarction in previous studies [3, 4]. Additionally, indicators of nutritional status and systemic inflammation, such as lymphocycte count, peripheral monocytosis and albumin levels were associated with an impaired outcome in patients with ST-elevation myocardial infarction (STEMI) [4, 5]. Prolonged reperfusion times further increase the likelihood of impaired left ventricular function [6, 7]. 

Over the last decades, substantial efforts have been made to improve outcomes of patients with myocardial infarction. These include enhancements in healthcare infrastructures, such the increase of cardiac catheterization laboratories and the development of hospital networks for pre-hospital triage to the appropriate institution, which have successfully reduced reperfusion times [8]. Additionally, advances in secondary preventative measure including lipid-lowering therapies and heart failure medication have substantially contributed to better patient outcomes [1]. 

In times of modern healthcare structures, reperfusion strategies and heart failure medication, our study investigated, which factors still impact 1-year left ventricular function in a vulnerable population of patients with STEMI as first-time presentation of coronary artery disease.

## Methods

### Study design and cohort

**Fig. 1:**
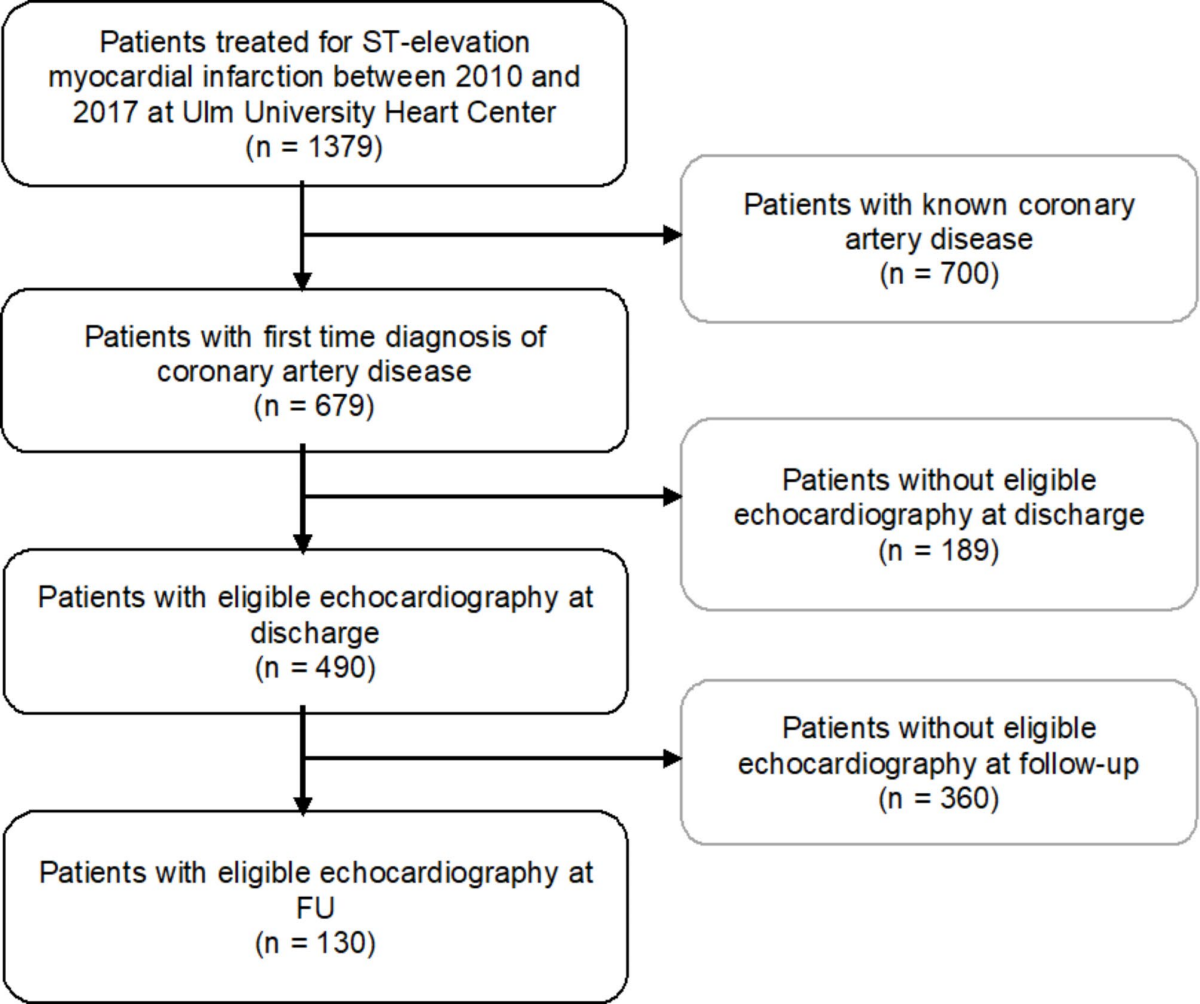
Flow diagram of patient inclusion

This retrospective, single-center study reviewed 1,379 consecutive patients diagnosed with STEMI and treated at the Ulm University Heart Center between January 2010 and December 2017. Patients were included if coronary artery disease was not previously diagnosed by coronary CT or cardiac catheterization, and if full-cycle echocardiography of 2- and 4-chamber view was available at discharge and at 12-months ± 3 months follow-up (FU) (see more Fig. 1). Ulm University Heart Center is a typical German tertiary hospital that serves in an area with a high density of catheterization laboratories, however, not all provide 24/7 service. Each patients’ residence was analyzed, and besides of 3 patients (1 patient presented directly in our emergency without emergency doctor, 1 patient was initially admitted due to another reason and 1 patient was in the region of our hospital), the maximal distance was ~ 40 km in our cohort. STEMI was defined in accordance with current guidelines [9]. Pain-to-balloon time was defined as the time between first clinical signs of the acute coronary syndrome and the first application of a balloon or direct stent implantation. Symptom onset time was obtained from emergency protocols, with the exact time or time range of symptom-onset was recorded. When only a time range was available, the exact onset was estimated based on protocols and patient anamnesis and the cohort´s pain-to-balloon time was divided into four quartile-based categories. Demographic, clinical and laboratory data were extracted from our patient management system. Laboratory data at admission was derived from blood controls prior or during cardiac catheterization for STEMI. Troponin T is routinely measured prior and post cardiac catheterization and at least 24 h daily until the peak Troponin T is observed. Peak troponin T was defined as the highest value of high-sensitivity cardiac troponin T during the hospital stay and baseline Troponin T as the value assessed from blood samples prior to cardiac catheterization. Troponin T was defined as the Troponin T value closest to discharge. Reduced LVEF ≤ 50% at 1-year follow-up was defined as the primary endpoint. Clinical trial number: not applicable. The investigation conforms with the principles outlined in the Declaration of Helsinki and was approved by the Ulm University ethics committee (369/21) on January 10th 2022.

### Left ventricular function assessment

Left ventricular function was semiautomatically assessed from the 2- and 4-chamber view using a commercially available software for offline left ventricular calculation (TOMTEC; TomTec Imaging Systems, Unterschleissheim, Germany) based on best consensus by two echocardiographic readers (D.F., S.F.). Echocardiography was performed using a Philips iE33 (Koninklijke Philips N.V., The Netherlands) diagnostic ultrasound machine. Following initial cardiac catheterization, the eligible echocardiography closest to discharge was chosen. For 1-year follow-up echocardiography, the closest eligible echocardiography at 12 months was chosen. Left ventricular function at admission was assessed by levocardiography and categorized semiquantitatively as normal (LVEF 50–70%), mild dysfunction (LVEF 40–49%), moderate dysfunction (LVEF 30–39%) and severe dysfunction (LVEF less than 30%). Cardiac catheterization and levocardiographic assessment were performed by experienced interventional cardiologists. Echocardiography prior to discharge and at 1-year follow-up were performed by experienced echocardiographers at our echocardiography laboratory in accordance with current guidelines [10]. 

### Statistical analysis

The study cohort was grouped according to LVEF ≤ 50% and > 50% at 1-year follow-up. Data are displayed as mean ± standard deviation, median with 25th – 75th percentiles or proportions (%). The distribution of continuous variables was tested with the Shapiro-Wilk test. Normally distributed variables were analyzed using the student’s t-test and non-normally distributed variables using the unpaired U-test. Categorial variables were compared by the chi-square test. Preinterventional variables potentially influencing LVEF at 12-month FU, defined as a p-value < 0.2 in Table 1 were further analyzed using univariate logistic regression analysis. Significantly tested variables from univariate logistic regression were included to the multiple logistic regression analysis. A sub-analysis of predictors of LVEF improvement to ≥ 50% at 1-year follow-up in patients with reduced LVEF < 50% at discharge was performed. (Suppplemental table S2). In the logistic regression analysis, the specific STEMI type under investigation was compared against all other regions. A P-value of < 0.05 was considered statistically significant and the statistical analysis was performed using IBM^®^ SPSS^®^ Statistics Version 29.

## Results

### Baseline and follow-up data

130 consecutive patients with STEMI as first-time presentation of coronary artery disease and eligible follow-up echocardiography were analyzed. LVEF was levocardiographically reduced in 94% patients at admission, and echocardiographically in 69% patients at discharge and in 45% patients at 1-year FU. Baseline, procedural and echocardiographic data of patients with LVEF < 50% at 1-year FU are displayed in Table 1. Patients with reduced FU LVEF suffered significantly more often from atrial fibrillation (12% in patients with reduced FU LVEF vs. 3%, *p* = 0.043) and anterior STEMI (59% vs. 23%). Pain-to-balloon time was categorized according to the median time (159 [111–247] minutes) in 4 quartiles: 0–111, 112–159, 160–246 and 247–784 min. Patients with reduced FU LVEF were more often in the upper quartile (35% vs. 17%), whereas patients with a FU LVEF ≥ 50% were more frequently in the lower quartile (3% vs. 41%). An explorative sub-analysis of the Pain-to-balloon time was added to the supplements (S1). All patients underwent revascularization. Postinterventional thrombolysis in myocardial infarction (TIMI) III flow did not differ between both groups (95% vs. 96%). Complete revascularization at discharge was comparable in both groups. Although no significant differences were observed in the periprocedural characteristics, there were consistent minor disparities to the disadvantage of the group with reduced LV function. For instance, a trend towards increased procedure duration, contrast volume, and radiation dose was observed indicating a more complex procedure. Additionally, the group had a 1.7% rate of TIMI I flow in the final angiographic recordings, whereas no TIMI I flow was observed in the comparison group. The use of angiotensin converting enzyme (ACE) inhibitors, Angiotensin II receptor blocker (AT I) blockers, and beta-blockers exceeded 90% and was comparable in both groups. The use of mineralocorticoid antagonist (MRAs) differed significantly, with a markedly higher rate in the group with LVEF < 50%, however, reaching approximately 40%. Median LVEF was 40 [35–45] vs. 51 [44–55] %, *p* < 0.001 at discharge and 43 [37–46] vs. 55 [52–58] %, *p* < 0.001 at 1-year FU.

**Table 1:** Consecutive patients undergoing cardiac catheterization for STEMI as first-time presentation of coronary artery disease between 2010 and 2017 stratified by LVEF < 50% and ≥ 50% at one-year follow-up

	LVEF < 50% at follow-up (*n* = 59)	LVEF ≥ 50% at follow-up (*n* = 71)	*P*-value
**Baseline characteristics**			
Male	46 (78.0%)	52 (73.2%)	0.533
Age, years	64 ± 13	62 ± 11	0.396
Height, cm	172 ± 9	171 ± 9	0.712
Weight, kg	83 [73–90]	80 [73–90]	0.615
BMI, kg/m^2^	27 [25–31]	28 [25–31]	0.905
Arterial hypertension	46 (78.0%)	48 (67.6%)	0.189
Diabetes mellitus	12 (20.3%)	6 (8.5%)	0.051
Hyperlipidemia	49 (83.1%)	57 (80.3%)	0.685
Smoking - Active - Former	22 (37.3%) 6 (10.2%)	30 (42.3%) 15 (21.1%)	0.108
Family history of cardiovascular diseases	16 (27.1%)	19 (26.8%)	0.963
Atrial fibrillation	7 (11.9%)	2 (2.8%)	**0.043**
Former lung embolism	2 (3.4%)	1 (1.4%)	0.454
Former stroke or TIA	5 (8.5%)	2 (2.8%)	0.155
Peripheral artery disease	2 (3.4%)	2 (2.8%)	0.851
COPD	5 (8.5%)	1 (1.4%)	0.056
Creatinine, µmol/l - Admission - Discharge	88 [77–105] 90 [80–102]	85 [72–95] 85 [71–93]	0.202 **0.013**
Troponin, ng/l - Admission - Peak - Discharge	567 [59–2553] 3610 [1765–5965] 2009 [927–3322]	221 [77–1598] 1766 [946–3707] 1157 [756–2219]	0.295 **0.005** **0.018**
NYHA class at admission - I - II - III - IV	32 (24.6%) 1 (0.8%) 4 (3.1%) 18 (13.5%)	35 (26.9%) 3 (2.3%) 6 (8.5%) 26 (20.0%)	0.446
**Procedural characteristics of STEMI treatment**			
Pain-to-balloon time quartiles, minutes 0–111 112–159 160–246 247–784	1 (2.7%) 10 (27.0%) 13 (35.1%) 13 (35.1%)	22 (40.7%) 13 (24.1%) 10 (18.5%) 9 (16.7%)	**< 0.001**
Coronary artery disease - 1-vessel - 2-vessel - 3-vessel	9 (15.3%) 16 (27.1%) 34 (57.6%)	17 (23.9%) 21 (29.6%) 33 (46.5%)	0.357
Main stem stenosis	9 (15.3%)	6 (8.5%)	0.227
Culprit vessel - LM/prox.LAD - LAD - CX - RCA	36 (61.0%) 52 (88.1%) 40 (67.8%) 41 (69.5%	37 (52.1%) 57 (80.3%) 37 (52.1%) 48 (67.6%)	0.308 0.226 0.070 0.818
STEMI type - anterior - posterior - lateral - posterolateral - anterolateral - multiple	35 (59.3%) 16 (27.1%) 2 (3.4%) 6 (10.2%) 0 0	24 (22.8%) 40 (56.3%) 1 (1.4%) 3 (4.2%) 2 (2.8%) 1 (1.4%)	**0.008**
Levocardiographic ventricular function - ≥ 50% - 41–49% - ≤ 40%	1 (1.7%) 9 (15.3%) 49 (83.1%)	7 (9.9%) 20 (28.2%) 44 (62.0%)	**0.019**
Complete revascularization at discharge	18 (30.5%)	25 (35.2%)	0.570
TIMI flow - I - II - III	1 (1.7%) 2 (3.4%) 56 (94.9%)	0 3 (4.2%) 68 (95.8%)	0.531
Periprocedural events - CPR - Ventricular fibrillation - Coronary perforation - Pericardial tamponade - Atrial fibrillation	3 (5.1%) 2 (3.4%) 0 0 4 (6.8%)	1 (1.4%) 1 (1.4%) 0 0 2 (2.8%)	0.227 0.454 - - 0.284
GPIIbIIIA-Inhibitor use	18 (30.5%)	17 (23.9%)	0.401
Realized access - Right femoral artery - Right radial artery - Both	2 (3.4%) 52 (88.1%) 2 (3.4%)	5 (7.0%) 63 (88.7%) 1 (1.4%)	0.513
Duration of PCI, minutes	54 [36–67]	46 [30–64]	0.114
Amount of contrast agent, ml	190 [160–195]	175 [150–190]	0.131
Radiation time, minutes	12 [8–17]	12 [8–18]	0.919
Radiation dose, Gray	75 [45–125]	62 [34–113]	0.339
Postprocedural data			
Total hospital stay, days	8 [6–10]	6 [6–7]	**< 0.001**
Medication at discharge - ASS - P2Y12-Inhibitor - DOAC - ACE/AT1-Inhibitor - Beta blockers - MRA - SGLT2-Inhibitor - Diuretics - Statin	57 (96.6%) 59 (100%) 7 (11.9%) 54 (91.5%) 58 (98.3%) 23 (39.0%) 0 24 (40.7%) 59 (100%)	69 (97.2%) 71 (100%) 5 (7.0%) 68 (95.8%) 66 (93.0%) 9 (12.7%) 1 (1.4%) 11 (15.5%) 71 (100%)	0.851 - 0.344 0.316 0.148 **< 0.001** 0.360 **0.001** -
P2Y12 inhibitor type at discharge - Clopidogrel - Prasugrel - Ticagrelor	14 (10.8%) 29 (22.3%) 16 (12.3%)	16 (12.3%) 43 (33.1%) 12 (9.2%)	0.310
**Echocardiographic data**			
LVEF at discharge, %	40 [35–45]	51 [44–55]	**< 0.001**
LVEF at 1-year follow-up, %	43 [37–46]	55 [52–58]	**< 0.001**
Days between STEMI and 1-year follow-up echocardiography	382 ± 59	384 ± 71	0.766
LVEF at 1-year follow-up - ≥ 40% and < 50% - < 40%	39 (66.1%) 20 (33.9%)	0 0	-
Class improvement at 1-year FU - Any - ≥ 40% and < 50% to > 50% - < 40% to > 50% - < 40% to ≥ 40% and < 50%	13 (22.0%) 0 0 13 (22.0%)	31 (43.7%) 22 (31.0%) 9 (12.7%) 0	**0.009** **< 0.001** **< 0.001** **0.005**
**1-year follow-up**			
Medication at follow-up - ASS - P2Y12-Inhibitor - NOAC - ACE/AT1-Inhibitor - Beta blockers - MRA - ARNI - SGLT2-Inhibitor - Diuretics - Statin	53 (89.8%) 29 (49.2%) 7 (11.9%) 53 (89.8%) 56 (94.9%) 26 (44.1%) 2 (3.4%) 0 21 (35.6%) 56 (94.9%)	68 (95.8%) 38 (53.5%) 7 (9.9%) 66 (93.0%) 64 (90.1%) 7 (9.9%) 1 (1.4%) 1 (1.4%) 12 (16.9%) 68 (95.8%)	0.184 0.620 0.713 0.524 0.309 **< 0.001** 0.454 0.360 **0.015** 0.816
One year heart failure hospitalization	2 (3.4%)	1 (1.4%)	0.454
NYHA class at 1-year follow-up - I - II - III - IV	39 (30.0%) 15 (11.5%) 3 (3.2%) 0	51 (39.2%) 18 (13.8%) 0 0	0.284

ARNI: angiotensin receptor-neprilysin inhibitor, ASS: acetylsalicylic acid, BMI: body mass index, COPD: chronic obstructive pulmonary disesase, CPR: cardiopulmonary resuscitation, CX: circumflex, DOAC: direct oral anticoagulant, eGFR: estimated glomerular filtration rate, LAD: left anterior descending, LM: left main, LVEF: left ventricular function, MRA: mineralocorticoid antagonist, PCI: percutaneous coronary intervention, STEMI: ST-elevation myocardial infarction, SGLT2: sodium glucose transporter 2, RCA: right coronary artery, TIA: transient ischemic attack, TIMI: thrombolysis in myocardial infarction, TVR: target vessel revascularization

P-values < 0.05 are presented bold. LVEF was semiautomatically assessed from the 2- and 4-chamber view

### Predictors of LVEF < 50% at 1-year follow-up

Table 2 presents univariate and multiple logistic regression of preinterventional variables of Table 1 potentially influencing LVEF < 50% at 1-year follow-up. Anterior STEMI significantly increased (OR 2.9 [95%-CI 1.4–5.8], *p* = 0.004) and posterior STEMI significantly decreased (OR 0.3 [95%-CI 0.1–0.6], *p* = 0.001) the likelihood of reduced FU LVEF in logistic regression analysis. Furthermore, the higher the quartile of pain-to-balloon time, the higher was the chance of a reduced FU LVEF (OR 16.9, *p* = 0.011; OR 28.6, *p* = 0.002; OR 31.8, *p* = 0.002). These observations persisted even after adjustment using multiple regression analysis: anterior wall myocardial infarction (OR 3.2 [95%-CI 1.2–6.9], *p* = 0.018) and pain-to-balloon time with each quartile (OR 15.7, *p* = 0.014; OR 30.8, *p* = 0.002; OR 33.7 *p* = 0.002). Binomial logistic regression model was statistically significant for the multiple logistic regression model: χ²(4) = 27.55, *p* < 0.001 and the Hosmer-Lemeshow test indicated adequate model quality: χ²(6) = 1.79, *p* = 0.938.

**Table 2:** Logistic regression analysis to identify predictors of LVEF ≥ 50% at follow-up

Univariate logistic regression analysis			
	Odds ratio	95%-Confidence interval	P-value
Diabetes mellitus	2.77	0.97–7.89	0.057
Arterial hypertension	1.69	0.77–3.74	0.191
Smoking	0.52	0.26–1.05	0.070
Atrial fibrillation	4.64	0.93–23.28	0.062
Former stroke or TIA	3.19	0.59–17.11	0.175
COPD	6.48	0.74–57.12	0.092
STEMI type - anterior - posterior	2.86 0.29	1.39–5.84 0.14–0.61	**0.004** **0.001**
Pain-to-balloon time quartiles, minutes 0–111 112–159 160–246 247–784	16.92 28.60 31.78	1.94–147.77 3.28–249.73 3.60–280.21	**0.011** **0.002** **0.002**
**Multiple logistic regression analysis**			
Anterior STEMI	3.19	1.19–8.59	**0.021**
Pain-to-balloon time quartiles, minutes 0–111 112–159 160–246 247–784	15.65 30.76 33.66	1.75–140.42 3.39–278.73 3.68–307.95	**0.014** **0.002** **0.002**

COPD: chronic obstructive pulmonary disesase, STEMI: ST-elevation myocardial infarction, TIA: transient ischemic attack

Preinterventional variables potentially influencing LVEF at 12-month FU, defined as a p-value < 0.2 in Table 1 were further analyzed using univariate logistic regression analysis

P-values < 0.05 are presented bold

## Discussion

This study aimed to evaluate factors contributing to 1-year left ventricular dysfunction in a distinct cohort of patients who experienced STEMI as the first clinical manifestation of coronary artery disease, even in the setting of modern healthcare structures. Anterior wall infarction and prolonged pain-to-balloon time independently increased the likelihood of LVEF < 50% at midterm follow-up. Both factors, acting independently of one another, underscore the importance of both infarct location and timely intervention in the management of STEMI, highlighting the persistent challenges in achieving optimal cardiac recovery in this patient population. In our study, LVEF was semiautomatically assessed by 2- and 4-chamber echocardiography at discharge and 1-year follow-up using the commercially available TOMTEC software for LVEF assessment further underscoring the validity of the assessed LVEF value.

Modern treatment of myocardial infarction includes timely primary PCI, secondary preventative treatments and heart failure medication, which have already led to reduced mortality in patients with myocardial infarction over the last decades [1, 11]. However, LV dysfunction persists in 22–44% of STEMI patients, depending on the population and treatments applied [2, 12]. Additionally, Chew et al. found that LV-reassessment at FU is not always performed, potentially affecting reported rates of maintained LV dysfunction at FU [12]. Early identification of patients with persistent LV dysfunction is crucial due to heightened risk of sudden cardiac arrest and potential benefits from preventative interventions, such as expanded heart failure medication or implantable cardiac defibrillator implantation [13–15]. In this study, 69% of patients had reduced LV function at discharge, compared to 94% at admission, and 45% still had reduced function at the 1-year follow-up in this specific STEMI population.

Besides comorbidities, peak troponin T, an indicator of myocardial ischemia remains a key predictor of persistent LVEF reduction and heart failure across several studies [2, 3]. Reduced LVEF and infarct scars are also associated with malignant arrhythmias [16]. Timely reperfusion is essential to mitigate these risks and prevent chronic heart failure, as delays exacerbate myocardial damage [6]. Reduction of time to reperfusion was achieved by structural improvements including hospital networks and technological improvements including artificial intelligence applications as recently demonstrated for the detection of occlusion myocardial infarction will further improve these times within healthcare structures [17, 18]. However, the symptom onset to first medical contact time is still crucial, as it potentially prolongs the time of myocardial ischemia and ultimately, left ventricular dysfunction.

Patients unfamiliar with heart related chest-pain are at increased risk, especially oligosymptomatic patients, who potentially misinterpret these symptoms. In this study, a delay of more than 2 h from symptom onset to reperfusion was strongly associated with a reduced LVEF < 50% at midterm follow-up, with the risk increasing significantly over time. This finding underscores the current ESC guideline, which recommends primary PCI-mediated reperfusion within 2 h of STEMI diagnosis [19]. It additionally emphasizes the critical need to minimize pain to first medical contact time to enable timely STEMI diagnosis.

Multiple regression analysis compellingly showed that each quartile increase in pain-to-balloon time substantially elevated the likelihood of reduced LVEF, with odds ratios of 15.7 (*p* = 0.014), 30.8 (*p* = 0.002), and 33.7 (*p* = 0.002), respectively. These findings underscore that near-time reperfusion starting with the first symptoms is crucial, even in an era of modern healthcare structures with treatment protocols, secondary preventative measures and heart failure medication. Further analysis of the pain-to-balloon time demonstrated prolonged door-to-balloon time in patients with impaired 1-year LVEF. However, different factors have an influence on this time including a complex arterial access, but also high procedural complexity indicated by longer procedure times in our data [20]. Notably, an even larger delay was observed in the pain-to-first medical contact time. Community education programs may help reduce these delays, as demonstrated by Wang et al., who found that such programs effectively shortened the time from symptom-onset to first medical contact [21]. Vulnerable groups, particularly those with a higher risk of myocardial infarction or prolonged symptom to first medical contact time, may benefit significantly from targeted interventions. For example, prolonged symptom onset to first medical contact time in women with acute coronary syndrome (ACS) was shown by Zhou et al. [22] In part, atypical symptoms in women with ACS contribute to this delay. Rodrigues et al. found that low income and diabetes are independent predictors of late presentation in STEMI patients, further emphasizing the need to address at-risk groups through tailored educational and healthcare strategies [23]. 

Even after adjusting for pain-to-balloon time, anterior wall myocardial infarction (AMI) still predicts reduced LVEF at midterm follow-up. Compared to other infarct locations, AMI is accompanied with a greater extent of myocardial damage and higher rates of persistent left ventricular dysfunction, as demonstrated by numerous studies [2, 3, 24]. 

This study highlights the ongoing challenges in optimizing care for patients with myocardial infarction, with the high incidence of persistent left ventricular dysfunction serving as a critical reminder of the gaps still present in current management. These findings should prompt further refinements in clinical protocols and community education efforts aimed at reducing treatment delays and ultimately improving patient outcomes.

## Limitations

The retrospective study design is associated with all the inherent limitations ascribed to this study type. Some emergency protocols only provided a time range rather than a specific time point for symptom onset. However, by categorizing pain-to-ballon-time into four realistic time periods and its results underline the well-established time-critical nature of STEMI treatment. Patients were only included if eligible echocardiography was available to assess LVEF with the well-validated Simpson method with eligible 2- and 4-chamber views at discharge and follow-up. During the study period, only a few patients were treated with ARNI (angiotensin receptor-neprilysin inhibitor) and SGLT-2 (sodium glucose transporter 2) inhibitors, which may potentially influence the LV-function at follow-up. Furthermore, the patient number is a limitation of this study.

## Conclusion

Given the vulnerability of patients with STEMI as their first presentation of coronary artery disease, especially those unfamiliar with heart-related chest pain, a differentiated treatment approach is necessary. Enhancing patient education, streamlining prehospital care, and a tailored clinical approach focusing on rapid diagnosis and primary PCI can further reduce reperfusion times and improve outcomes.

## Electronic supplementary material

Below is the link to the electronic supplementary material.

Supplementary Material 1

## Data Availability

The datasets used and/or analysed during the current study are available from the corresponding author on reasonable request.

## Abbreviations

ACE: Angiotensin converting enzyme
ASS: Acetylsalicylic acid
AT 1: Angiotensin II receptor blocker
AI: Artificial intelligence
BMI: Body mass index
COPD: Chronic obstructive pulmonary disesase
CPR: Cardiopulmonary resuscitation
CI: Confidence interval
CX: Circumflex
DOAC: Direct oral anticoagulant
eGFR: Estimated glomerular filtration rate
FU: Follow-up
HR: Hazard ratio
LAD: Left anterior descending
LM: Left main
LVEF: Left ventricular ejection fraction
PCI: Percutaneous coronary intervention
MRA: Mineralocorticoid antagonist
OR: Odds ratio
RCA: Right coronary artery
SGLT2: Sodium glucose transporter 2
STEMI: ST-elevation myocardial infarction
TIA: Transient ischemic attack
TIMI: Thrombolysis in myocardial infarction
TVR: Target vessel revascularization
