# Supplementary material for: Prolonged pain-to-balloon time still impairs midterm left ventricular function following STEMI
Source: BMC Cardiovasc Disord. 2025 Jan 23;25:37. doi: 10.1186/s12872-025-04484-3 (PMC11756106; doi:10.1186/s12872-025-04484-3)
Supplement: Supplementary file 1 — Supplementary Material 1 [file 12872_2025_4484_MOESM1_ESM.docx]

Supplements

| **Table S1** Consecutive patients undergoing cardiac catheterization for STEMI as first-time presentation of coronary artery disease between 2010 and 2017 stratified by LVEF <50% and ≥50% at one-year follow-up | | | |
| --- | --- | --- | --- |
|  | LVEF < 50%  at follow-up  (n = 59) | LVEF ≥ 50%  at follow-up  (n = 71) | P-value |
| Pain to first medical contact, minutes | 79 [36 – 210] | 35 [11 – 150] | **0.043** |
| First medical contact to door, minutes | 42 [25 – 53] | 41 [35 – 61] | 0.286 |
| Door to balloon time, minutes | 56 [37 -79] | 39 [29 - 63] | **0.013** |
| LVEF: left ventricular function; STEMI: ST-elevation myocardial infarction  P-values <0.05 are presented bold. | | | |

| **Table S2** Predictors of 1-year LVEF <50% in patients with reduced LVEF at discharge following hospitalization for STEMI | | | |
| --- | --- | --- | --- |
| **Univariate logistic regression analysis** | | | |
|  | Odds ratio | 95%-Confidence interval | P-value |
| Male | 0.69 | 0.26 – 1.86 | 0.464 |
| Age, years | 1.01 | 0.98 – 1.05 | 0.447 |
| Height, cm | 1.16 | 0.01 – 176.41 | 0.953 |
| Weight, kg | 1.01 | 0.97 – 1.04 | 0.760 |
| BMI, kg/m^2^ | 1.02 | 0.92 – 1.12 | 0.733 |
| Arterial hypertension | 1.45 | 0.54 – 3.89 | 0.464 |
| Diabetes mellitus | 3.70 | 0.77 – 17.39 | 0.102 |
| Hyperlipidemia | 0.94 | 0.29 – 3.05 | 0.921 |
| Smoking | 0.49 | 0.20 – 1.22 | 0.126 |
| Family history of cardiovascular diseases | 1.28 | 0.46 – 3.53 | 0.639 |
| Atrial fibrillation | - | - | 0.999 |
| Former lung embolism | 1.05 | 0.09 – 12.09 | 0.967 |
| Former stroke or TIA | 2.78 | 0.31 – 24.89 | 0.361 |
| Peripheral artery disease | 1.05 | 0.09 – 12.09 | 0.967 |
| COPD | - | - | 0.999 |
| Creatinine, µmol/l   - Admission - Discharge | 1.01  1.02 | 0.99 – 1.03  0.99 – 1.04 | 0.180  0.088 |
| Troponin, ng/l   - Admission - Peak - Discharge | 1.00  1.00  1.00 | 1.00 – 1.00  1.00 – 1.00  1.00 – 1.00 | 0.998  0.395  0.348 |
| NYHA class at admission   - I - II - III - IV | 0.20  0.81  0.56 | 0.02 – 2.44  0.13 – 4.99  0.22 – 1.47 | 0.209  0.823  0.241 |
| Pain-to-balloon-time quartiles, minutes  0 – 111  112 – 159  160 – 246  247 - 784 | 16.67  43.33  32.50 | 1.69 – 164.79  3.89 – 481.82  3.13 – 337.81 | **0.016**  **0.002**  **0.004** |
| Coronary artery disease   - 1-vessel - 2-vessel - 3-vessel | 1.58  2.16 | 0.45 – 5.54  0.69 – 6.73 | 0.475  0.185 |
| Main stem stenosis | 1.68 | 0.42 – 6.72 | 0.463 |
| Culprit vessel   - LM/prox.LAD - LAD - CX - RCA | 0.86  0.79  1.97  1.44 | 0.35 – 2.12  0.19 – 3.21  0.81 – 4.81  0.58 – 3.58 | 0.745  0.754  0.135  0.434 |
| STEMI type   - anterior - posterior | 1.77  0.39 | 0.74 – 4.26  0.16 – 0.99 | 0.202  **0.046** |
| Complete revascularization at discharge | 0.92 | 0.36 – 2.35 | 0.865 |
| Periprocedural events   - CPR - Ventricular fibrillation - Coronary perforation - Pericardial tamponade - Atrial fibrillation | 1.61  1.05  -  -  1.06 | 0.16 – 16.13  0.09 – 12.09  -  -  0.18 – 6.11 | 0.687  0.967  -  -  0.953 |
| GPIIbIIIA-Inhibitor use | 1.83 | 0.64 – 5.22 | 0.259 |
| Duration of PCI, minutes | 0.96 | 0.89 – 1.03 | 0.233 |
| Total hospital stay, days | 1.19 | 0.99 – 1.42 | 0.053 |
| Medication at discharge   - ASS - P2Y12-Inhibitor - DOAC - ACE/AT1-Inhibitor - Beta blockers - MRA - SGLT2-Inhibitor - Diuretics - Statin | -  -  -  0.73  -  1.84  -  3.57  - | -  -  -  0.136 – 4.08  -  0.70 – 4.79  -  1.20 – 10.59  - | -  -  **-**  0.734  -  0.214  **-**  **0.022**  - |
| P2Y12 inhibitor type at discharge   - Clopidogrel - Prasugrel - Ticagrelor | 0.89  0.69  1.94 | 0.33 – 2.44  0.29 – 1.68  0.63 – 5.91 | 0.828  0.422  0.246 |
| ASS: acetylsalicylic acid, BMI: body mass index, COPD: chronic obstructive pulmonary disease, CPR: cardiopulmonary resuscitation, CX: circumflex, DOAC: direct oral anticoagulant, eGFR: estimated glomerular filtration rate, LAD: left anterior descending, LM: left main, LVEF: left ventricular function, MRA: mineralocorticoid antagonist, PCI: percutaneous coronary intervention, STEMI: ST-elevation myocardial infarction, SGLT2: sodium glucose transporter 2, RCA: right coronary artery, TIA: transient ischemic attack, TIMI: thrombolysis in myocardial infarction, TVR: target vessel revascularization  P-values <0.05 are presented bold. | | | |
